# Performance Evaluation and Optimization of an Ink/Polyurethane Actuator for Light-Driven Soft Gripper

**DOI:** 10.3390/polym17223004

**Published:** 2025-11-12

**Authors:** Quanwang Niu, Xiangyu Gu, Hao Wu, Weiyang Yu, Xiaohong Yan, Xiangfu Wang

**Affiliations:** 1 College of Electronic and Optical Engineering & College of Flexible Electronics (Future Technology), Nanjing University of Posts and Telecommunications, Nanjing 210023, China; 2024020311@njupt.edu.cn (Q.N.); 1223024904@njupt.edu.cn (X.G.); 1222025019@njupt.edu.cn (H.W.); 2 School of Physics and Electronic Information, Henan Polytechnic University, Jiaozuo 454003, China; yuweiyang@hpu.edu.cn

**Keywords:** actuators, light control, photothermal effect, reliability

## Abstract

Flexible light-driven actuators are emerging as critical components for next-generation soft robotics and intelligent systems, demanding simple fabrication and robust performance. This work details the optimization and characterization of a flexible, low-cost light-driven actuator based on an ink/polyurethane bilayer composite. We maximized the actuation performance, including bending angle and response speed, by systematically tuning the ink concentration and polyurethane layer thickness. Optimal results were achieved at an ink concentration of 220 mg/mL and a polyurethane thickness of 50 µm, which thermal analysis confirmed is due to an ideal balance between photothermal efficiency and mechanical integrity. The actuator also exhibited excellent durability, showing no performance degradation after 500 continuous cycles. Our results validate this material system for applications like soft grippers and offer clear design principles for enhancing photothermal actuator performance.

## 1. Introduction

Traditional robots, primarily composed of rigid materials, have played a significant role in industrial automation. However, their limitations—such as bulky size, poor environmental adaptability, high energy consumption, and costly fabrication—have become increasingly apparent. In recent years, soft robots, with their flexible materials and innovative design principles, have emerged as a rapidly advancing field. The swift development of soft robots is attributed to progress in materials science, intelligent actuation technologies, and bionics. The fundamental component of a soft robot is the soft actuator, and as material science has advanced, the range of materials for fabricating these actuators has diversified significantly. Key materials now include carbon-based materials [1,2,3], hydrogels [4,5,6], liquid crystal elastomers (LCEs) [7,8], and shape-memory polymers [9], all chosen for the high performance they enable due to their unique physical and chemical properties. Similarly, actuation methods are highly varied, with common approaches including light [10], temperature [11], humidity [12], electric fields [13], magnetic fields [14], and pH [15]. Among these, light-driven soft actuators stand out because light-driven methods offer distinct advantages, including remote control, high immunity to electromagnetic interference, and versatile modulation options [16]. This allows them to perform complex motions, eliminating the need for cumbersome external control systems. This capability is particularly crucial in environments requiring flexible manipulation.

Polyurethane (PU) is an ideal material for fabricating light-driven soft actuators due to its excellent mechanical properties, good wear resistance, and elasticity. As a polymer with highly tunable properties, PU’s physical characteristics can be modified by altering its molecular structure, hardness, and degree of cross-linking. In recent years, researchers have conducted extensive studies on PU-based actuators, developing devices such as underwater biomimetic actuators [17], water-surface “motors” [18], and light-driven smart grippers and crawling robots [19]. Furthermore, Chinese ink has a history spanning thousands of years, widely employed in calligraphy and brush painting due to its excellent stability, strong adhesion [20], low cost, and ready availability [21]. Recently, with the advancement of flexible electronics, the carbon materials within Chinese ink have been identified as key components for achieving electrical conductivity and superior photothermal conversion efficiency [22,23,24,25]. This has led to the exploration of ink in various novel functional devices, for which PU often serves as an essential flexible substrate. For instance, Wei et al. utilized a surface swelling technique to uniformly coat carbon particles from Chinese ink onto mask lanyards (PU/polyester blended fibers). The resulting ink coating formed a conductive network, enhancing the sensitivity of the sensor [22]. Similarly, Lin et al. developed a reprogrammable bilayer light-driven actuator based on Chinese ink and PU, which also successfully integrated a sensing function based on the ink film’s thermal resistance [26]. The primary actuation principle of these ink/PU light-driven actuators relies on the mismatch in the coefficients of thermal expansion (CTE) between the layers. The ink film exhibits strong light absorption, generating significant heat under near-infrared (NIR) irradiation. This heat is transferred to the adjacent layer. Crucially, the PU layer possesses a high positive CTE, while the ink film is reported to have a negative CTE. Consequently, upon heating, the PU layer expands, whereas the ink film contracts. This pronounced asymmetric deformation forces the composite actuator to bend toward the ink layer.

Evidently, studying the thermal and mechanical properties of PU-based photothermal materials under NIR irradiation is crucial for designing effective light-driven soft actuators and robots. Therefore, this paper focuses on evaluating the actuation performance and reliability of a NIR light-driven soft actuator based on an ink/PU composite, and exploring its applicability for light-driven gripper applications. Specifically, we fabricated a series of soft actuators by regulating the ink concentration and the thickness of the PU layer. We then tested their temperature changes and bending angles during deformation to identify the optimal performance parameters. Furthermore, we assessed their anti-fatigue properties. Finally, we demonstrated the light-driven grasping of a cylindrical object using the fabricated soft actuator.

## 2. Materials and Methods

### 2.1. Materials

PU solution (F0410) was purchased from Shenzhen Jitian Chemical Co., Ltd. (Shenzhen, China). It is a single-component resin emulsion with low volatile organic compound content. The solution cures at either room temperature or with heating, forming a transparent and hydrophilic PU film. Table 1 shows the detailed performance index. The ink, a traditional Chinese calligraphy ink, was purchased from Beijing Yidege Ink Co., Ltd. (Beijing, China). Its primary components are water, carbon black, and animal bone glue. Upon evaporation of the water, the animal bone glue and carbon black form a homogeneous film [27].

### 2.2. Design and Fabrication of Actuators

**Figure 1:**
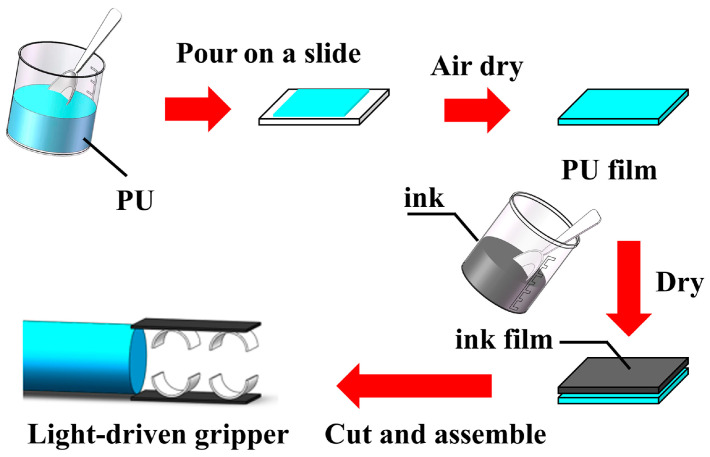
Preparation process of the ink/PU light-driven actuator and gripper.

The fabrication process for the ink/PU light-driven actuator is illustrated in Figure 1. Initially, the glass substrate was pre-cleaned using an ultrasonic cleaner. A uniform PU film was then prepared by spin-coating a PU solution onto the substrate. The PU solutions were prepared by mixing the stock PU solution with deionized water to achieve various concentrations (220, 280, 350, 400, and 510 mg/mL) to produce films of different thicknesses (50, 70, 90, 110, and 130 µm). A low spin speed of 200 rpm was used to allow the PU solution to level evenly on the glass surface, followed by drying at 25 °C for 24 h. Subsequently, ink solutions were prepared by vigorously mixing the ink with deionized water for 10 min until fully homogeneous. The ink concentrations were adjusted to 160, 180, 200, 220, and 240 mg/mL, respectively. This solution was then poured onto the dried PU film, and the sample was dried again at 25 °C for 24 h. This process formed a bilayer ink/PU composite with a tightly bonded ink layer. Finally, the desired ink/PU light-driven actuators and grippers were obtained by precisely cutting and assembling this composite film.

### 2.3. Characterization

Photothermal actuation was investigated using two light sources: a 980 nm semiconductor infrared laser (MW-GX-980, Changchun Laser Optoelectronics Technology Co., Ltd., Changchun, China) and an NIR lamp (Philips BR125, Signify, Eindhoven, The Netherlands). During irradiation, an infrared thermal camera (HM-TPK20-3AQF/W, Hikvision Digital Technology Co., Ltd., Hangzhou, China) was used to record real-time temperature changes and map the temperature distribution across the actuator’s surface. Photographs of the physical samples were taken at 25 °C using a smartphone (iPhone 14 Pro, Apple Inc., Cupertino, CA, USA). Scanning electron microscope (SEM) images were obtained using a microscope (Inspect F50, FEI Company, Hillsboro, OR, USA) operating in High Vacuum mode with a spot size of 2.0 (SE detector). The optical absorbance of the ink and PU films was characterized using a UV–VIS–NIR spectrophotometer (UV-3600, SHIMADZU, Kyoto, Japan).

## 3. Results and Discussion

### 3.1. Photomechanical Bending Characterization

**Figure 2:**
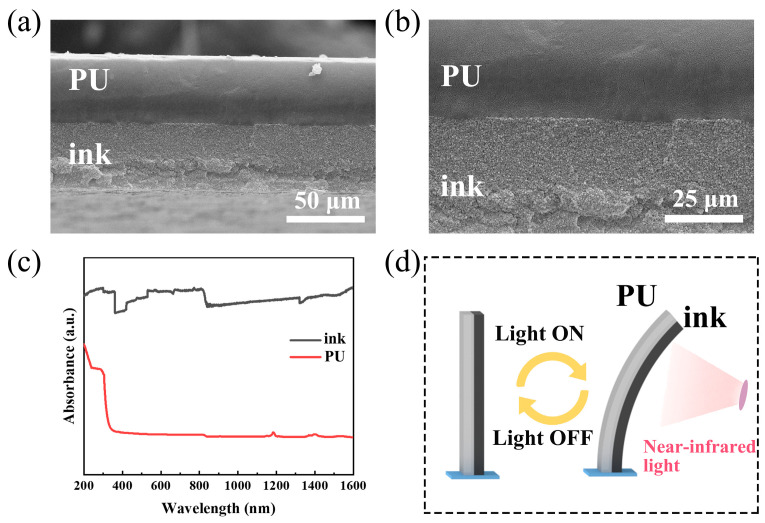
(**a**,**b**) SEM image of the ink/PU composite film; (**c**) absorption spectra of ink and PU; (**d**) schematic diagram of the deformation of the ink/PU light actuator.

Figure 2 shows the cross-sectional SEM images of the ink/PU composite film at different magnifications. The low-magnification image in Figure 2a clearly displays the overall bilayer structure, consisting of a smooth, dense upper PU layer and a rougher lower ink layer composed of aggregated carbon nanoparticles. Figure 2b, a high-magnification view of the interface, confirms that the two layers are tightly bonded. The boundary is distinct, with no visible gaps or delamination, indicating excellent adhesion between the ink and PU layers. Figure 2c shows the absorption spectra of the ink and PU films. The ink film exhibits strong light absorption across the entire wavelength range, whereas the PU film shows pronounced absorption only in the ultraviolet region (200–400 nm) and minimal absorption in the NIR region. Since the NIR photons absorbed by the ink layer are primarily dissipated through nonradiative relaxation, a significant photothermal effect can be generated by NIR irradiation. This results in localized heating of the ink layer, which establishes a temperature gradient across the ink/PU interface. Due to the opposite signs of the CTE (negative for the ink and positive for the PU), the PU film expands and the ink film shrinks. The bilayer bends toward the ink side when illuminated with NIR light, as illustrated in Figure 2d. When the NIR light is turned off, the temperature gradient disappears as the heat dissipates into the environment and the actuator returns to its initial flat state.

**Figure 3:**
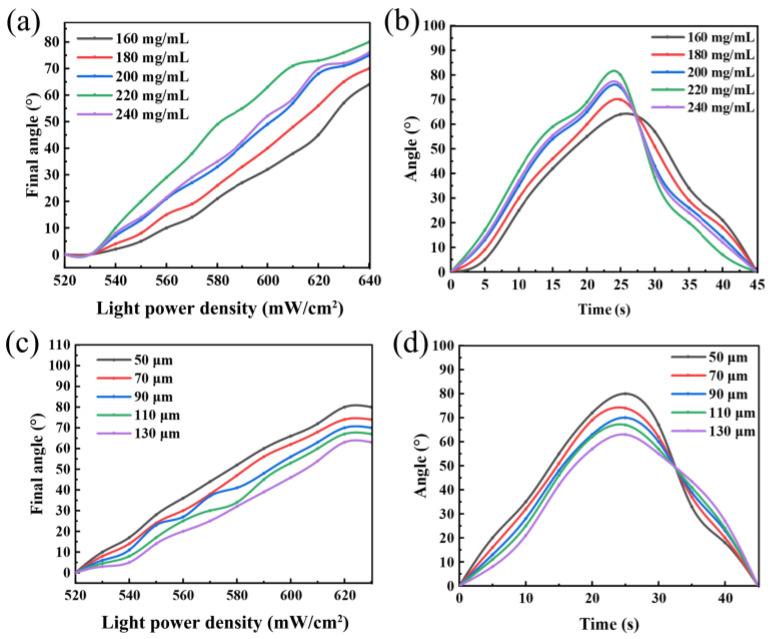
Influence of fabrication parameters on the photothermal actuation performance: (**a**,**b**) effect of ink concentration on actuation: (**a**) final bending angle as a function of laser power density and (**b**) the corresponding bending angle evolution over time; (**c**,**d**) effect of PU layer thickness on actuation: (**c**) final bending angle as a function of laser power density and (**d**) the corresponding bending angle evolution over time.

To optimize the performance of the actuator, the effects of ink concentration and PU layer thickness were systematically investigated. First, the influence of ink concentration was examined. For this study, a constant PU thickness of 50 µm was utilized, as this value provides a practical balance between flexibility and the structural integrity required for robust device fabrication. Figure 3a plots the final bending angles of actuators with various ink concentrations as a function of increasing laser power density. While all samples showed increased bending with higher power density, optimal performance was observed at an ink concentration of 220 mg/mL. Increasing the concentration further to 240 mg/mL did not yield further improvement, likely due to increased material stiffness. This optimum is also reflected in the actuation speed analysis (Figure 3b). At a fixed power density of 620 mW/cm^2^, the actuator with 220 mg/mL ink achieved a maximum bending angle of 80° in the shortest time (~23 s). Therefore, an ink concentration of 220 mg/mL was identified as optimal and used in subsequent experiments.

Next, using the optimal ink concentration, the effect of PU layer thickness was studied. Figure 3c displays the final bending angles for actuators with PU thicknesses ranging from 50 µm to 130 µm. A clear inverse relationship was observed: the thinner the PU layer, the greater the bending angle. The 50 µm actuator achieved the largest bend of approximately 80°, while the angle progressively decreased to ~64° for the 130 µm film. This trend is corroborated by the response speed (Figure 3d), where the 50 µm actuator was the fastest (~22 s) and the 130 µm actuator was the slowest (>28 s). These results collectively demonstrate that a thinner PU layer significantly enhances both the magnitude and speed of actuation.

### 3.2. Photothermal Conversion Analysis

**Figure 4:**
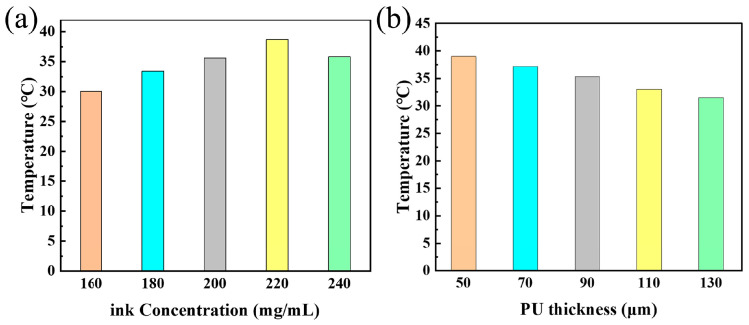
Influence of fabrication parameters on the photothermal performance of the actuators: (**a**) peak temperature versus ink concentration; (**b**) peak temperature versus PU layer thickness. All measurements were performed under a constant laser power density of 620 mW/cm^2^.

Figure 4a displays the maximum temperatures achieved as a function of ink concentration. This temperature profile directly mirrors the actuation performance shown in Figure 3a, with a peak temperature of nearly 40 °C occurring at the optimal ink concentration of 220 mg/mL. This confirms that this concentration provides the most efficient light-to-heat conversion. Lower concentrations likely result in insufficient light absorption, while higher concentrations may increase material stiffness and alter thermal conductivity, both of which can impair the final actuation.

**Figure 5:**
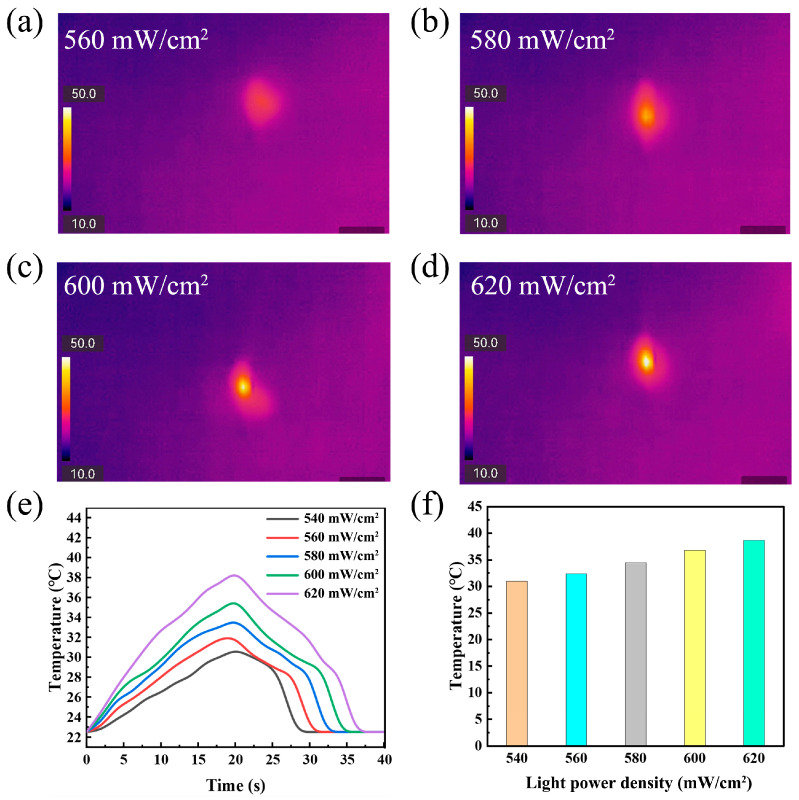
Photothermal characterization of the actuator: (**a**–**d**) infrared thermal images displaying the temperature distribution under irradiation at 560, 580, 600, and 620 mW/cm^2^, respectively; (**e**) real-time temperature profiles of the actuator at various power densities; (**f**) plot of peak temperature versus applied laser power density.

Similarly, the thermal data explains the effect of actuator thickness (Figure 4b). A clear inverse relationship between PU thickness and maximum temperature was observed, with the thinnest (50 µm) actuator reaching nearly 40 °C, while the thickest (130 µm) only attained 32 °C. This result confirms that thinner films, possessing lower thermal mass, heat up more efficiently. This superior thermal response directly translates to the faster actuation speeds and larger bending angles observed previously (Figure 3c,d). The photothermal response of the optimized actuator (220 mg/mL ink, 50 µm PU) was then characterized as a function of laser power density (Figure 5). The infrared thermal images (Figure 5a–d) and quantitative plots (Figure 5e,f) consistently demonstrate that higher power densities lead to higher steady-state temperatures and faster heating rates. Specifically, the maximum temperature increased from ~31 °C at 540 mW/cm^2^ to ~40 °C at 620 mW/cm^2^. This positive correlation confirms that the actuation effect can be precisely tuned by modulating the light intensity. However, it was noted that power densities exceeding 650 mW/cm^2^ caused irreversible damage to the film, defining the upper operational limit for the system.

### 3.3. Durability and Cyclic Stability

**Figure 6:**
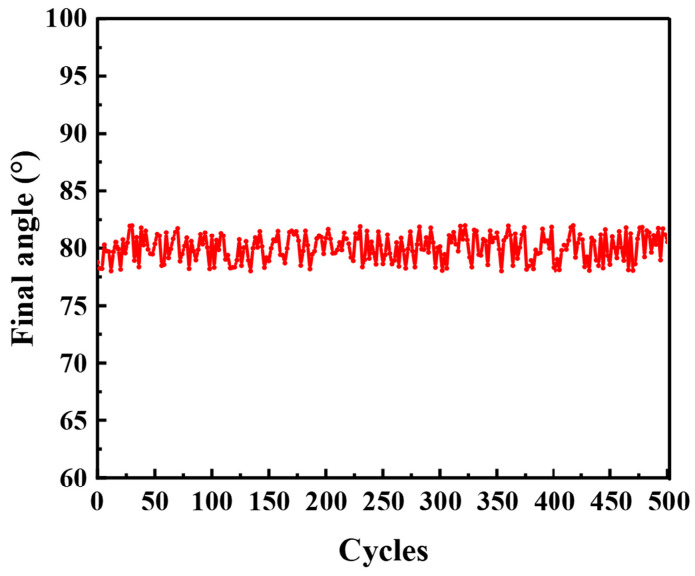
Repeatability test of the ink/PU actuator.

To assess its operational stability and long-term durability, the optimized actuator (50 µm PU, 220 mg/mL ink) was subjected to a cyclic actuation test. The test consisted of 500 repeated cycles of actuation under 620 mW/cm^2^ NIR light, followed by complete recovery upon light removal. As shown in Figure 6, the actuator consistently achieved its maximum bending angle of approximately 80° throughout all cycles, with no observable performance degradation. This result confirms the excellent cycling stability and mechanical robustness of the actuator, highlighting its potential for reliable, long-term applications.

### 3.4. Application as a Light-Driven Soft Gripper

**Figure 7:**
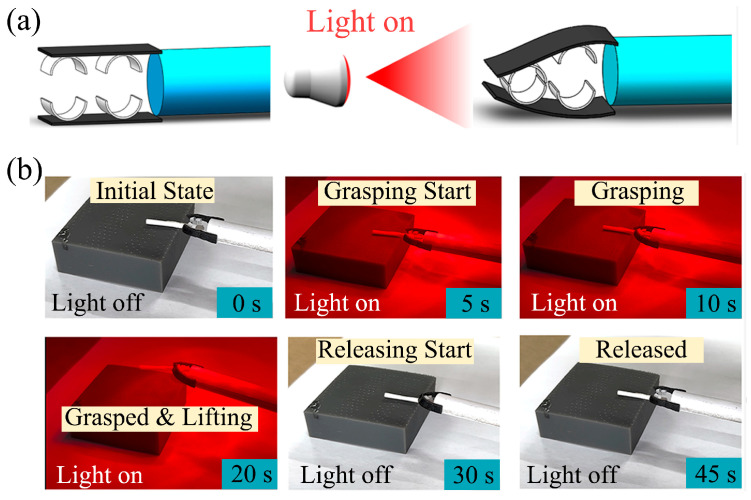
(**a**) The structure and action process of the light-driven gripper based on ink/PU; (**b**) a grasping process is completed by the gripper under the drive of NIR light.

To construct the light-driven gripper, two ink/PU actuators were arranged in an opposing configuration. The ink layers were oriented inward to face each other, while the PU layers faced outward. To enhance gripping stability, four arc-shaped polyethylene pads were affixed to the inner surface of the ink layers. The resulting gripper assembly is depicted in Figure 7a.

The dynamic process of object grasping and releasing was demonstrated, as illustrated in Figure 7b. A paper rod was used as the test object. In its initial state, the two actuator arms of the gripper were parallel. Upon 5 s of NIR light irradiation (the emission spectrum is shown in Figure S1), the actuators exhibited significant inward bending. After 20 s of continuous exposure, the gripper had firmly secured the target object, enabling it to be lifted. Following the cessation of the NIR light, the gripper began to relax, initiating the release of the object at the 30 s mark. The object was fully released by 45 s, corresponding to 25 s after the light was turned off, completing the entire actuation cycle.

To quantitatively evaluate the gripper’s grasping capability, test loads (paper rods) were used, starting from 100 mg and incrementing by 20 mg. The actuator could successfully lift and stably hold a maximum load of approximately 180 mg. Above this weight, the actuator still exhibited a grasping motion but could not fully lift the object. Given that the light-driven gripper has a total mass of 35 mg, this performance demonstrates a lifting capacity approximately 5.1 times its own weight. This result confirms the feasibility of the designed ink/PU composite for practical applications in light-driven soft robotics.

However, it is noteworthy that the grasping speed of this light-driven actuator is relatively slow compared to pneumatic or electric flexible systems [28,29]. This limitation is primarily attributed to the inherently slow processes of photothermal conversion and subsequent thermal diffusion. To further enhance the response speed, future work could explore photochemical approaches. Utilizing materials that undergo rapid phase transitions, rather than relying on photothermal effects, could induce faster macroscopic motion. Thus, the primary advantage of this photothermal actuator over electric or pneumatic systems is not speed, but its capacity for remote energy delivery. This enables the wireless control of soft robots in specialized scenarios, such as transporting components within strong electric-field environments or sampling in toxic atmospheres. Furthermore, to enhance functionality, the light-driven platform could be expanded by developing multi-stimuli-responsive materials. Integrating responses to humidity, pH, or ambient gas concentrations would enable intelligent, multi-signal interactions between the material and its environment.

## 4. Conclusions

In conclusion, this work demonstrates the successful optimization of a low-cost, light-driven ink/PU soft actuator. By tuning the ink concentration to 220 mg/mL and the PU thickness to 50 µm, this optimization resulted in a large bending angle (~80°), responsive actuation, and excellent stability over 500 cycles. The practical viability of this photothermal system was validated by a proof-of-concept soft gripper capable of lifting over five times its own mass.

## Figures and Tables

**Table 1:** PU solution performance index.

Performance Index	pH (25 °C)	Viscosity (mPa·s)	Film Forming Temperature Conditions (°C)	Storing Temperature (°C)
Index value	6∼8	800∼1200	20	5∼30

## Data Availability

The original contributions presented in this study are included in the article/Supplementary Material. Further inquiries can be directed to the corresponding authors.

## Supplementary Materials

The following supporting information can be downloaded at: https://www.mdpi.com/article/10.3390/polym17223004/s1, Figure S1: Emission spectrum of the light source to drive the soft gripper.
