# Supplementary material for: Performance Evaluation and Optimization of an Ink/Polyurethane Actuator for Light-Driven Soft Gripper
Source: Polymers (Basel). 2025 Nov 12;17(22):3004. doi: 10.3390/polym17223004 (PMC12656543; doi:10.3390/polym17223004)
Supplement: Supplementary file 1 [file polymers-17-03004-s001.zip › polymers-3966219-supplementary.pdf]

Supplementary files

Performance Evaluation and Optimization of an Ink/Polyurethane Actuator for Light-Driven Soft Gripper

Quanwang Niu [1], Xiangyu Gu [1], Hao Wu [1], Weiyang Yu [2], Xiaohong Yan [1,*] and Xiangfu Wang [1,*]

[1] College of Electronic and Optical Engineering & College of Flexible Electronics (Future Technology), Nanjing University of Posts and Telecommunications, Nanjing 210023, China; 2024020311@njupt.edu.cn (Q.N.); 1223024904@njupt.edu.cn (X.G.); 1222025019@njupt.edu.cn (H.W.)

[2] School of Physics and Electronic Information, Henan Polytechnic University, Jiaozuo 454003, China; yuweiyang@hpu.edu.cn

* Correspondence: yanxh@njupt.edu.cn (X.Y.); xfwang@njupt.edu.cn (X.W.)

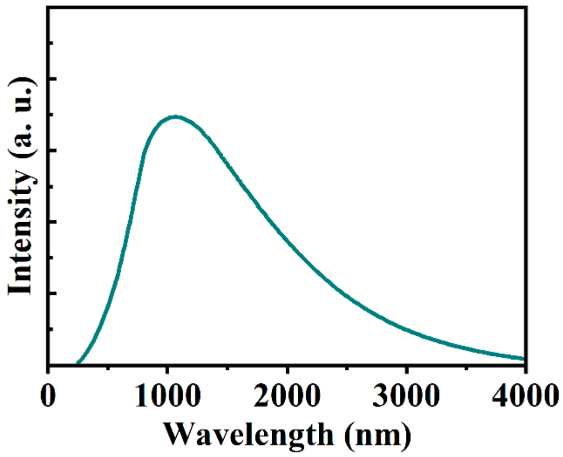

Figure S1. Emission spectrum of the light source to drive the soft gripper.
